# Structural basis for the pathogenicity of parkin catalytic domain mutants

**DOI:** 10.1016/j.jbc.2024.108051

**Published:** 2024-12-03

**Authors:** Julian P. Wagner, Véronique Sauvé, Anshu Saran, Kalle Gehring

**Affiliations:** 1 Department of Biochemistry, McGill University, Montreal, Quebec, Canada; 2 Centre de Recherche en Biologie Structurale, McGill University, Montreal, Quebec, Canada

**Keywords:** PRKN, parkin, E3 ubiquitin ligase, ubiquitin, structural biology, AlphaFold

## Abstract

Mutations in the E3 ubiquitin ligase parkin cause a familial form of Parkinson’s disease. Parkin and the mitochondrial kinase PTEN-induced kinase 1 assure quality control of mitochondria through selective autophagy of mitochondria (mitophagy). Whereas numerous parkin mutations have been functionally and structurally characterized, several Parkinson’s disease mutations found in the catalytic Rcat domain of parkin remain poorly understood. Here, we characterize two pathogenic Rcat mutants, T415N and P437L. We demonstrate that both mutants exhibit impaired activity using autoubiquitination and ubiquitin vinyl sulfone assays. We determine the minimal ubiquitin-binding segment and show that both mutants display impaired binding of ubiquitin charged on the E2 enzyme. Finally, we use AlphaFold 3 to predict a model of the phospho-parkin:phospho-ubiquitin:ubiquitin-charged E2 complex. The model shows the repressor element of parkin and the N-terminal residues of the catalytic domain form a helix to position ubiquitin for transfer from the E2 to parkin. Our results rationalize the pathogenicity of the parkin mutations and deepen our understanding of the active parkin:E2∼Ub complex.

Parkinson’s disease (PD) is the second most common neurodegenerative disease and characterized by a set of clinical symptoms including motor symptoms, for instance tremors, rigidity and postural instability, and nonmotor symptoms such as dysphagia or hyposmia. On a cellular level, a hallmark of PD is the loss of dopaminergic neurons in the *substantia nigra pars compacta*. Whereas most cases are sporadic, 5 to 15% of patients exhibit a familial form of the disease with Mendelian inheritance. A few highly penetrant mutations in different genes have been identified, among them mutations in PTEN-induced kinase 1 (*PINK1*) and *PRKN*. Biallelic pathogenic *PRKN* variants are the most common forms of autosomal recessive early-onset PD (1, 2). Together, they control a quality control system for autophagy of damaged mitochondria (termed mitophagy) (3).

The gene *PRKN* encodes the E3 ubiquitin (Ub) ligase parkin composed of a regulatory ubiquitin-like domain (Ubl) and a catalytic core of four zinc-binding domains: RING0, RING1, in-between-RING, and Rcat. (Fig. 1*A*). Parkin is a member of the RING-between-RING (RBR) family of E3 enzymes (4) that share a common two-step catalytic mechanism. Following binding of the Ub-charged Ub-conjugating enzyme (E2∼Ub), Ub is first transferred to the active site cysteine in the catalytic Rcat domain in a transthiolation reaction. Ub is then transferred to a lysine residue on a substrate protein (5). Parkin activity is tightly regulated and, in the absence of activation, adopts an autoinhibited conformation. Intradomain contacts between Rcat and RING0 block the catalytic cysteine, and the Ubl domain and a repressor element of parkin (REP) in the in-between-RING-Rcat linker prevent E2∼Ub binding (6).

**Figure 1:**
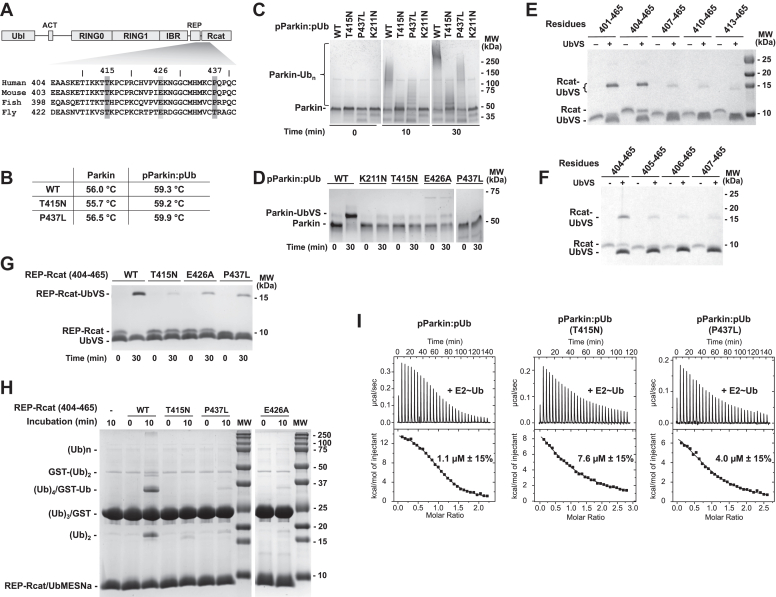
***In vitro* characterization of parkin Rcat mutants.***A*, parkin domain topology and sequence alignment of human, mouse, fish, and fly parkin around the T415N and P437L mutations. *B*, comparison of melting temperatures of inactive and activated WT, T415N, and P437L parkin. *C*, autoubiquitination assays of full-length activated WT, K211N, T415N, and P437L parkin. *D*, UbVS assays of full-length WT, K211N, T415N, E426A, and P437L phosphorylated parkin (pParkin) in the presence of pUb. *E* and *F*, determination of the minimal ubiquitin binding region using the UbVS assay with Rcat constructs of different length and (+) 30 min incubation time with UbVS. *G*, bypass assays with WT, T415N, E426A, and P437L parkin (residues 404–465). *H*, UbVS assays with WT, T415N, E426A, and P437L parkin (residues 404–465). *I*, isothermal titration calorimetry (ITC) experiments of WT, T415N, and P437L phosphorylated parkin in the presence of pUb titrated with UBE2L3 C86K charged with ubiquitin through an isopeptide bond to prevent E2 discharging during the experiment. The mutants showed a reduced affinity for E2∼Ub. pUb, phospho-ubiquitin; UbVS, ubiquitin vinyl sulfone.

Parkin is activated by PINK1, a protein kinase that accumulates on damaged mitochondria, where it phosphorylates Ub forming phospho-ubiquitin (pUb). This phosphorylation event recruits parkin to mitochondria through pUb-binding by the RING1 domain. This triggers the release of Ubl from RING1 facilitating a second phosphorylation event in which the parkin Ubl domain is phosphorylated by PINK1. This induces large scale conformational changes in parkin (7, 8, 9, 10, 11, 12), freeing the catalytic Rcat domain and initiating substrate ubiquitination. Ubiquitination of mitochondrial proteins initiates selective autophagy of the damaged mitochondria. How this protects against Parkinson’s disease (PD) is an active area of research (3).

Parkin activation has been studied in detail with multiple structures of inactive and active conformations determined (13, 14, 15, 16, 17, 18). However, less is known about how parkin interacts with the Ub-charged E2 enzyme (E2∼Ub); there are no structures of the active complex with E2∼Ub that allow identification of the critical interactions for Ub transfer. Structures of other RBR E3 ligases with E2∼Ub bound have been published, including HHARI:UBE2L3∼Ub (19, 20), HOIP:UBE2D2∼Ub (21), HOIL-1:Ub:UBE2L3∼Ub, and RNF216:Ub:UBE2L3∼Ub (5). These complexes revealed a conserved transthiolation complex structure specific to the RBR catalytic mechanism in which the E2 and E3 catalytic cysteines are aligned for the direct transfer of Ub. Interactions between the RBR domain and the E2∼Ub stabilize its open conformation to promote the transthiolation reaction (22). The interactions between Rcat and E2∼Ub are mainly mediated by Ub itself (5).

Here, we present biochemical and biophysical characterization of two parkin PD mutations, T415N and P437L, located in the catalytic domain of parkin. We determine the minimal catalytic domain that binds Ub and use AlphaFold 3 to predict the structure of the complex of phosphorylated parkin (pParkin), pUb, and UBE2L3∼Ub. The computational model provides a structural explanation for the pathogenicity of T415N and P437L parkin mutations and the mechanism of Ub transfer. Understanding the full range of structural conformations of parkin and its pathogenic variants will be essential for designing drugs targeting parkin.

## Results and discussion

### Functional characterization of parkin mutants T415N and P437L and identification of the minimal functional Rcat domain

Functional and structural characterization of parkin enabled researchers to better understand and classify benign and pathogenic variants (23, 24). However, despite these efforts, the defect in some disease variants remains unclear. Here, we focused on two single amino acid substitutions in the catalytic Rcat domain, T415N and P437L (Fig. 1*A*) (25). Their pathogenicity cannot be explained by the available parkin structures. Neither is located at positions obviously implicated in stability, zinc ion coordination, catalytic activity, or activation. Previous *in cellulo* studies showed that, following mitochondrial damage from carbonyl cyanide 3-chlorophenylhydrazone treatment, both variants cause an impairment of mitophagy compared to WT parkin (23).

We first evaluated the thermal stability of the T415N and P437L parkin mutants to rule out the possibility that the mutations affect the stability of the protein. The mutants did not show any significant shifts in melting temperature (T_m_), neither in their inactive nor in their activated forms (Figs. 1*B* and S1). This is in agreement with the equal levels of expression observed in cell-based studies and a deep mutational scanning study (23, 26). We next confirmed the previously reported limited autoubiquitination activity of T415N and P437L mutants (Fig. 1*C*) (27, 28). Both mutants were strongly impaired with the P437L mutant showing slightly more activity than T415N. The PD mutant K211N with a disrupted pUbl/pUb-binding site was used as a negative control.

We used a ubiquitin vinyl sulfone (UbVS) assay to assess parkin activation (15, 29, 30). The assay uses a chemically reactive Ub derivative (31) to measure exposure of the Rcat catalytic cysteine. Activated WT parkin was fully crosslinked by UbVS, while only a faint crosslinked band can be seen for the T415N, P437L, and an additional Rcat mutant E426A (Fig. 1*D*). Glu426 is highly conserved (Fig. 1*A*) and is required for activity in *Drosophila* (32). There are two possible explanations for the lack of crosslinking. It is possible that the mutations prevent parkin activation such that the active cysteine remains buried at the RING0-Rcat interface. This is the case for the K211N mutant. Alternatively, the mutations could prevent binding between Ub and the Rcat domain, which is necessary for crosslinking. Experiments with vinyl sulfone derivatives of NEDD8 and SUMO showed these Ub-like molecules did not form adducts with fully activated pParkin:pUb or the free Rcat domain (Fig. S2).

To distinguish between these possibilities, we investigated the activity of the catalytic domain in the absence of the inhibitory RING0 domain. We repeated the UbVS assay using the REP-Rcat segment of parkin with different N-termini to determine the minimal catalytic fragment. The Rcat domain (residues 413–465) alone was not sufficient to permit UbVS crosslinking (Fig. 1*E*), whereas crosslinking was observed for longer fragments. Systematically shortening the fragments allowed us to determine parkin residues 404 to 465 to be the minimal fragment able to form crosslinks (Fig. 1*F*). This fragment was used in subsequent experiments.

### T415N and P437L mutations impair Ub binding to the parkin catalytic domain

We repeated the UbVS assay with the minimal construct to evaluate the effects of the Rcat variants. We saw reduced crosslinking with T415N, E426A, and P437L compared to the WT suggesting a loss of affinity for Ub (Fig. 1*G*). To confirm this, we used a different chemical probe, ubiquitin sodium 2-mercaptoethanesulfonate (Ub-MESNa), in a bypass assay that allows the Rcat domain to ubiquitinate substrates without E1 and E2 enzymes (33). We observed that all three Rcat mutants were severely limited in their ability to ubiquitinate glutathione-*S*-transferase (GST) or form di-Ub (Fig. 1*H*). In both UbVS and Ub-MESNa assays, the E426A and P437L variants showed a trace of activity, while the T415N variant was completely inactive.

To confirm that the mutations affect Ub binding, we carried out isothermal titration calorimetry (ITC) experiments (Fig. 1*I*). The titrations were conducted with activated (phosphorylated and pUb-bound) parkin and Ub covalently attached to UBE2L3 C86K *via* an isopeptide bond to prevent catalysis. In agreement with previous measurements (10), WT pParkin:pUb-bound UBE2L3∼Ub with micromolar affinity. Titrations with the T415N and P437L parkin mutants showed decreases in K_D_ of 7.6-fold and 4-fold, respectively. The larger effect of T415N mutation agrees with its larger effect in biochemical assays. In the related RBR E3 ligase, HHARI, the equivalent of the T415N mutation was shown to decrease both ligase activity and Ub binding (22).

### AlphaFold model of the pParkin:pUb:E2∼Ub complex

**Figure 2:**
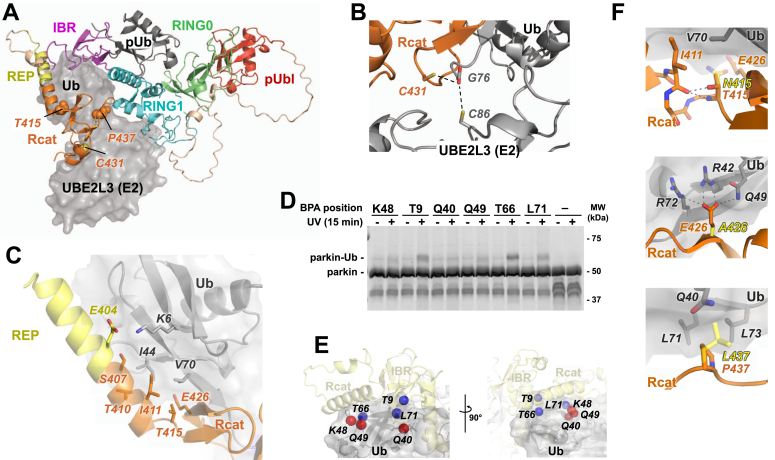
**AlphaFold 3 model of the activated parkin complex with a ubiquitin-charged E2.***A*, overall conformation of the predicted model of phosphorylated parkin with bound allosteric phospho-ubiquitin and ubiquitin-charged UBE2L3. An extension of the REP helix positions parkin Rcat domain above the ubiquitin-charged E2. *B*, zoom on the catalytic centers shows the parkin and E2 catalytic cysteines primed for transthiolation reaction (*dotted lines*). *C*, zoom on the ubiquitin interacting helix. The interaction interface is largely hydrophobic centered around ubiquitin residue I44 and V70. *D*, SDS-PAGE analysis of *p*-benzoyl-L-phenylalanine (BPA) crosslinking assays. Crosslinks were detected between phospho-parkin and ubiquitin molecules with BPA incorporated at their residues 9, 66, or 71. Ubiquitin without BPA reagent was used as a negative control (−). *E*, position of the six ubiquitin residues modified with the BPA crosslinking reagent in the AlphaFold 3 model of pParkin:pUb with E2∼Ub. Positions that generated crosslinks are colored *blue*; those that did not are colored *red*. *F*, modeling of PD mutations T415N, P437L, and the E426A substitution showing loss of hydrophobic interactions and side chains clashes, providing a structural explanation for the loss of catalytic activity. REP, repressor element of parkin.

We used AlphaFold 3 (34) to predict a computational model of the fully activated human pParkin:pUb:E2∼Ub complex (Fig. 2, *A–C*). The five predicted models were highly similar with strong quality metrics (ipTM and pTM scores >0.7 and favorable predicted aligned error (PAE) plot), suggesting a reliable prediction (Fig. S3). A similar AlphaFold model was recently reported by Connelly *et al.* (35). UbE2L3 binds RING1, pUb binds RING1, and pUbl binds RING0 as in the crystal structure of pParkin:pUb:E2 (18). Globally, the conformation of the complex is similar to related E3:E2∼Ub complexes with the RBR module wrapping around the E2∼Ub (5). The transthiolation center is of particular interest (Fig. 2*B*). The parkin catalytic cysteine is positioned next to the UBE2L3 catalytic cysteine in close vicinity of the C-terminal glycine of Ub enabling its transfer from UBE2L3 to parkin. This is similar to transthiolation centers observed in other RBR:E2∼Ub structures (5).

We next aimed to experimentally validate the structural model. We employed a *p*-benzoyl-L-phenylalanine (BPA) crosslinking assay (36). Upon irradiation with UV light, this noncanonical amino acid, incorporated at a specific position in a protein, can form covalent bonds with activated C-H bonds located within a radius of ∼6 Å. We introduced BPA at six positions (T9, Q40, K48, Q49, T66, and L71) on Ub charged on UBE2L3 and formed the pParkin:pUb:UBE2L3∼Ub complex. Analysis of the crosslinking reactions on reducing SDS-PAGE revealed the formation of parkin-Ub bands when Ub was modified at residues T9, T66, or L71 (Fig. 2*D*). In our AlphaFold model, those Ub residues are closer to parkin residues than the other three Ub sites (Fig. 2*E*). Attempts to determine the parkin residues crosslinked by mass spectrometry were unsuccessful, but the BPA experiment is consistent with the model and, together with other indicators described above, strengthens confidence in the AlphaFold model.

As in other RBR:E2∼Ub structures, a key interaction with the E2∼Ub is mediated by Ub on the E2 enzyme and an α-helix in parkin formed by the REP and the N-terminal residues of the Rcat domain (Fig. 2*C*). In inactive parkin, the REP promotes autoinhibition through binding RING1; however, upon activation, the REP detaches and forms a continuous α-helix with the Rcat to position Ub for the transthiolation reaction. Similar Ub guide helices have been reported for other RBR E3 ligases including parkin (35), RNF216 and HOIL-1 (5), HOIP (21), and HHARI (37). The minimal Ub-binding fragment (residues 404–465) determined in UbVS assays starts in the helix. Residue E404 is highly conserved and contacts Ub K6 (Fig. 2*C*). I411 interacts with the canonical Ub hydrophobic patch formed by I44 and V70 (Fig. 2*C*). Charge reversal of Ub K6 and parkin mutations S407G, T410S, and I411A in the Ub-binding helix were recently shown to prevent or diminish E2∼Ub discharging (35).

Additional residues in the Rcat domain stabilize Ub binding and maintain it in a position favoring the transthiolation reaction (Fig. S4). The model also reveals interactions between the Ub and UBE2L3 that maintain E2∼Ub in the open conformation and position the C-terminus of the Ub next to the parkin catalytic cysteine. Two aromatic C-terminal Rcat residues, W462 and F463, stack against a flat loop of UBE2L3 (res. 118–120) to maintain E2 and Rcat proximity for the Ub transfer (Fig. S5).

### Structural basis for the pathogenicity of the Rcat domain mutants

We used our AlphaFold model of the activated parkin complex to understand the structural consequences of the parkin mutants (Fig. 2*F*). While not part of the Ub-binding helix, residues T415, E426, and P437 all contact Ub. The side chain of T415 forms a hydrogen bond with the backbone of I411, stabilizing the guide helix. The T415N mutation forces the hydrophilic side chain of asparagine into the Ub V70 hydrophobic patch and creates close contacts with E426. E426 was previously shown to be essential for ubiquitination activity and its NMRs are sensitive to mutagenesis of T415 and Ub binding (32, 35). The negatively charged side chain of E426 forms extensive electrostatic interactions with Ub residues that are absent in the E426A mutant. Finally, modeling the P437L mutation predicts a steric clash of the large isobutyl sidechain of leucine with Ub Q40 while preventing hydrophobic interactions of the proline with Ub L71 and L73.

In conclusion, our biochemical assays and AlphaFold modeling reveal that the early-onset PD mutations T415N and P437L impair parkin ligase activity by disrupting Ub binding to the catalytic domain. The mutational effects are more pronounced in biochemical assays than binding assays, which likely reflects the importance of the conformation of the transthiolation complex. In the AlphaFold model, the parkin REP and Rcat form a guide helix that maintains the E2∼Ub in an open conformation and positions the catalytic cysteines together. These insights into the structure of the fully activated parkin in complex with an Ub-charged E2 enzyme are a valuable resource for future studies and bring us closer to finding an effective treatment for patients.

## Experimental procedures

### Cloning, expression, and purification of recombinant proteins in *Escherichia coli*

Single-point mutations and deletions were generated using PCR mutagenesis (Agilent) and proteins expressed in BL21 (DE3) *E. coli*. Purification of full-length and truncated parkin, UBE2L3, Tc-PINK1, and human His-E1 were done using methods described previously (7, 38). Briefly, proteins were purified by glutathione-Sepharose (Cytiva) or nickel-nitrilotriacetic acid agarose (Qiagen) affinity chromatography, followed by either 3C protease cleavage to remove the GST tag or Ulp protease cleavage to remove the His-SUMO tag. Size-exclusion chromatography was used as a last step. pParkin and pUb were produced and purified according to published procedures (29, 30). Purified proteins were verified using SDS-PAGE analysis. Protein concentrations were determined using UV absorbance.

### Autoubiquitination assays

The autoubiquitination assays of full-length WT and mutated parkin were performed at 22 °C for 10 or 30 min by adding 2 μM full-length phosphorylated WT, K211N, T415N, or P437L parkin in complex with pUb, to 100 nM human His-Ube1, 2 μM UBE2L3, 75 μM Ub in 50 mM Tris–HCl pH 8.0, 150 mM NaCl, 1 mM tris(2-carboxyethyl)phosphine (TCEP), 5 mM ATP, and 10 mM MgCl_2_. Reactions were stopped by the addition of reducing SDS-PAGE loading buffer and the level of ubiquitination was analyzed on SDS-PAGE gels stained with Coomassie blue.

### Differential scanning fluorimetry

To assess the thermal stability of the parkin mutants, 5 μM of nonphosphorylated or pParkin in complex with pUb were mixed with 1× Protein Thermal Shift Dye kit (Applied Biosystems, Life Technologies) in 30 mM Tris–HCl, 150 mM NaCl, and 1 mM TCEP, pH 8.0. The samples were heated from 25 to 99 °C in 1% steps using StepOnePlus (Applied Biosystems, Life Technologies). Data were analyzed using Protein Thermal Shift software 1.4 (Life Technologies). The maximum change of fluorescence with respect to temperature was used to determine the denaturation temperature. Experiments for each sample were performed in triplicate.

### UbVS assays

For UbVS assays, 2 μM parkin (either full-length or truncated parkin) were incubated in the presence of 10 μM UbVS (R&D Systems) in 30 mM Tris–HCl, 150 mM NaCl, 1 mM TCEP, pH 8.0 for 30 min at 37 °C. Reactions were stopped by the addition of reducing SDS–PAGE loading buffer, and the level of crosslinking was analyzed on SDS–PAGE gels (Bio-Rad) stained with Coomassie blue.

### Bypass assay

The bypass assay was adapted from reference (33). Ub-MESNa was prepared by incubation of 100 μM Ub, 250 nM His-E1, 100 mM MESNa, 10 mM MgCl_2_, and 10 mM ATP at 37 °C for 5 h in 0.1 M NaPO_4_, pH 8.0. The reaction was stopped by dialysis against 20 mM Hepes, 120 mM NaCl, pH 6.5. The Ub-MESNa was further purified by gel filtration in 20 mM Hepes, 120 mM NaCl, and 0.5 mM TCEP, pH 7.4. Assays of WT and mutated REP-Rcat (residues 404–465) were performed at 22 °C for 10 min by adding 1 μM WT, T415N, E426A, or P437L REP-Rcat, to 30 μM Ub-MESNa and 30 μM GST protein in 30 mM Hepes, 150 mM NaCl, 0.5 mM TCEP, pH 8.0. Reactions were stopped by the addition of reducing SDS-PAGE loading buffer, and the level of ubiquitination of GST was analyzed on SDS-PAGE gels stained with Coomassie blue.

### AlphaFold modeling

The structure of the pUb-bound phospho-parkin in complex with Ub-charged E2 enzyme was predicted using the AlphaFold server (Google DeepMind and Isomorphic Labs: https://alphafoldserver.com/, powered by AlphaFold 3) (34). The query consisted of the amino acid sequence of human parkin with phosphorylated serine 65, an Ub with phospho-serine at position 65, human UBE2L3 (E2 enzyme), an unmodified Ub and eight zinc ions (Zn^2+^). The prediction was run using standard settings, that is, five models were predicted and corresponding PAE plots produced. Models were visualized using The PyMOL Molecular Graphics System, Version 3.0.2 Schrödinger, LLC (https://pymol.org) and PAE plots were visualized using a custom python script.

### Preparation of UBE2L3 C86K charged with Ub through an isopeptide bond

A protocol for the Ub charging of UBE2D1 C85K (39) was adapted for UBE2L3 C86K to generate the UBE2L3∼Ub conjugate. UBE2L3 C86K (300 μM) was incubated with His-SUMO–tagged Ub (900 μM) and His_6_-UBE1 (30 μM) at 37 °C for 19 h in a buffer containing 100 mM Tris–HCl pH 10.0, 200 mM NaCl, 5 mM ATP, 10 mM MgCl_2_, and 0.8 mM TCEP. The reaction was diluted to 25 ml with binding buffer (50 mM Tris–HCl pH 8.0, 150 mM NaCl, 10 mM imidazole pH 8.0, and 3 mM ßME) and applied onto a nickel-nitrilotriacetic acid column (Qiagen). The column was washed with binding buffer and the E2∼Ub conjugate was eluted with elution buffer (50 mM Tris–HCl pH 8.0, 150 mM NaCl, 300 mM imidazole pH 8.0, and 3 mM ßME). The eluate was diluted with binding buffer and the His-SUMO tag from Ub was cleaved overnight at 4 °C with Ulp protease. The cleaved sample was applied onto a Superdex 75 gel-filtration column (Cytiva) connected to a His-Trap column (Cytiva), in 30 mM Tris–HCl pH 8.0, 150 mM NaCl, 10 mM imidazole pH 8.0, and 1 mM TCEP. The purified UBE2L3∼Ub conjugate was buffer exchanged into the ITC buffer.

### Isothermal titration calorimetry

ITC measurements were carried out at 20 °C using VP-ITC (Microcal). Samples were in 50 mM Tris–HCl, 150 mM NaCl, 1 mM TCEP, pH 7.4. Proteins (10 μM WT, T415N, or P437L pParkin:pUb) were titrated with one injection of 5 μl, followed by 28 injections of 10 μl of 100 μM or 88 μM UBE2L3 C86K charged with Ub. Data were fitted to a single set of identical sites using Origin v7 software.

### BPA crosslinking experiments

For expression of BPA-labeled Ub, the pEVOL-pBpF (Addgene plasmid #31190) and a GST-Ub plasmid were coexpressed in *E. coli* BL21 (DE3) in M9 minimal medium. The pEVOL-pBpF plasmid allows expression of a tRNA enabling the incorporation of the non-natural amino acid BPA in response to the amber codon, TAG. Expression of the tRNA was induced by adding 0.2% arabinose and 1 mM BPA, while GST-Ub expression was induced with 250 μM IPTG. The BPA-containing Ub was then purified using glutathione-Sepharose (Cytiva) affinity chromatography, followed by 3C protease cleavage to remove the GST tag. Subsequently, size-exclusion chromatography was carried out. Concentration was checked by UV absorbance and samples were sent to mass spectrometry to assess incorporation of BPA and degree of contamination by protein prematurely terminated due to incorporation of a STOP codon at TAG. To charge the E2 enzyme with the BPA-containing Ub, 100 nM human His-UBE1, 60 μM UBE2L3 with 4 mM ATP, and 8 mM MgCl_2_ were mixed and added to 200 μM Ub-BPA in 30 mM Tris–HCl pH 8.0, 150 mM NaCl, and 1 mM TCEP. The mixture was incubated for 1 h at 37 °C. Successful charging of UBE2L3 was checked by SDS-PAGE. pParkin C431A:pUb was added to a final concentration of 5 μM. Samples were UV-radiated at 365 nm for 15 min at 4 °C or kept at 4 °C without irradiation as a negative control. DTT-containing SDS-PAGE loading buffer was added to the samples before their analysis on SDS-PAGE gels stained with Coomassie blue.

## Data availability

All data described in this study are contained within the main article.

## Supporting information

This article contains supporting information (16).

## Conflict of interest

The authors declare that they have no conflicts of interest with the contents of this article.
