# Supplementary material for: Structural basis for the pathogenicity of parkin catalytic domain mutants
Source: J Biol Chem. 2024 Dec 3;301(1):108051. doi: 10.1016/j.jbc.2024.108051 (PMC11742612; doi:10.1016/j.jbc.2024.108051)
Supplement: Supplemental Figs. S1–S5 and Table S1 [file mmc1.pdf]

Supplementary material for

Structural Basis for the Pathogenicity of Parkin Catalytic Domain Mutants

Julian P. Wagner[1,2,*], Véronique Sauvé[1,2,*], Anshu Saran[1,2,†], Kalle Gehring[1,2]

[1]Department of Biochemistry, McGill University, Montreal QC H3G0B1, Canada

[2]Centre de Recherche en Biologie Structurale, McGill University, Montreal QC H3G0B1, Canada

[†]Present address: Department of Anatomy and Cell Biology, McGill University, Montreal, QC H3A 0C7, Canada.

*Equal contribution

Address for correspondence: Kalle Gehring, Dept. of Biochemistry, Life Science Complex, 3649 Prom. Sir-William-Osler, McGill University, Montreal QC H3G 0B1 Canada

Supplementary Table 1. Isothermal titration calorimetry data collection and fitting

Cell sample	Syringe sample	χ^2/DoF	Number of sites	Ka (M^{-1})	ΔH (cal/mol)	ΔS (cal/ mol/deg)
WT pS65 human parkin: pS65 Ub (10 μM)	UBE2L3 C86K~Ub (88 μM)	2.37E4	1.060 ± 0.008	9.06E5 ± 4.1E4	1.529E4 ± 160	79.4
T415N pS65 human parkin: pS65 Ub (10 μM)	UBE2L3 C86K~Ub (100 μM)	9591	0.961 ± 0.053	1.31E5 ± 1.1E4	1.724E4 ± 1.30E3	82.2
P437L pS65 human parkin: pS65 Ub (10 μM)	UBE2L3 C86K~Ub (100 μM)	1.591E4	0.986 ± 0.037	2.50E5± 2.5E4	9464 ± 513	57.0

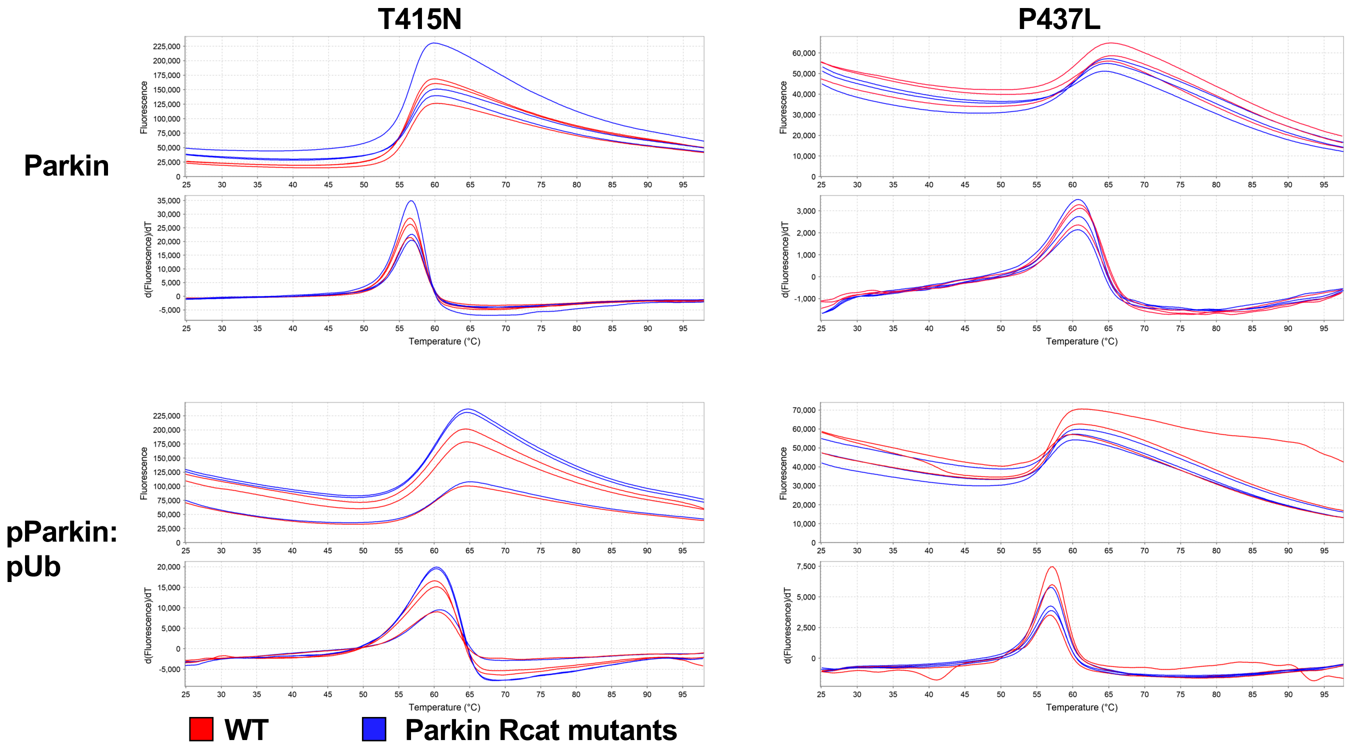

Supplementary Figure 1: The mutations T415N and P437L in the parkin Rcat domain do not affect protein stability in the autoinhibited or activated (phosphorylated and pUb-bound) condition. Thermal shift assays were carried out as described in methods. Derivative plot of replicates of the change in fluorescence vs. temperature. Since the assays for the mutants were performed on different days, new wild-type melting curves were generated. The melting temperatures (T_m) were within the expected margin of error and were averaged for the table comparing the T_m in figure 1D.

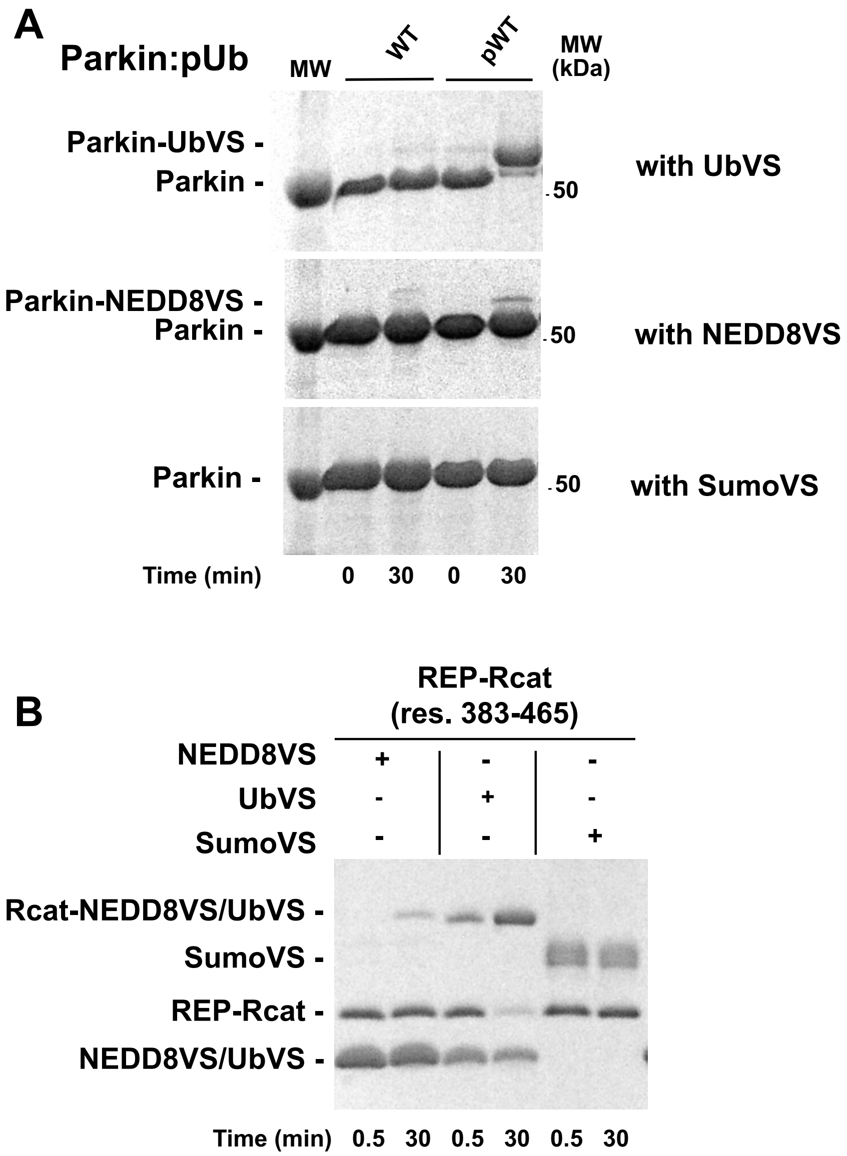

Supplementary Figure 2: Vinyl sulfone crosslinking is specific for activated parkin and for ubiquitin. (A) Non-phosphorylated (unactivated) and phosphorylated (activated) full-length parkin was crosslinked with different VS reagents (Ub, NEDD8, Sumo) and analyzed by SDS-PAGE. No crosslinks were observed without parkin activation and with VS probes other than ubiquitin. (B) Crosslinking assays with the isolated parkin Rcat domain confirm the specificity for UbVS. The small amount of crosslinking detected for NEDD8VS in both assays likely reflects the strong similarity of NEDD8 and ubiquitin.

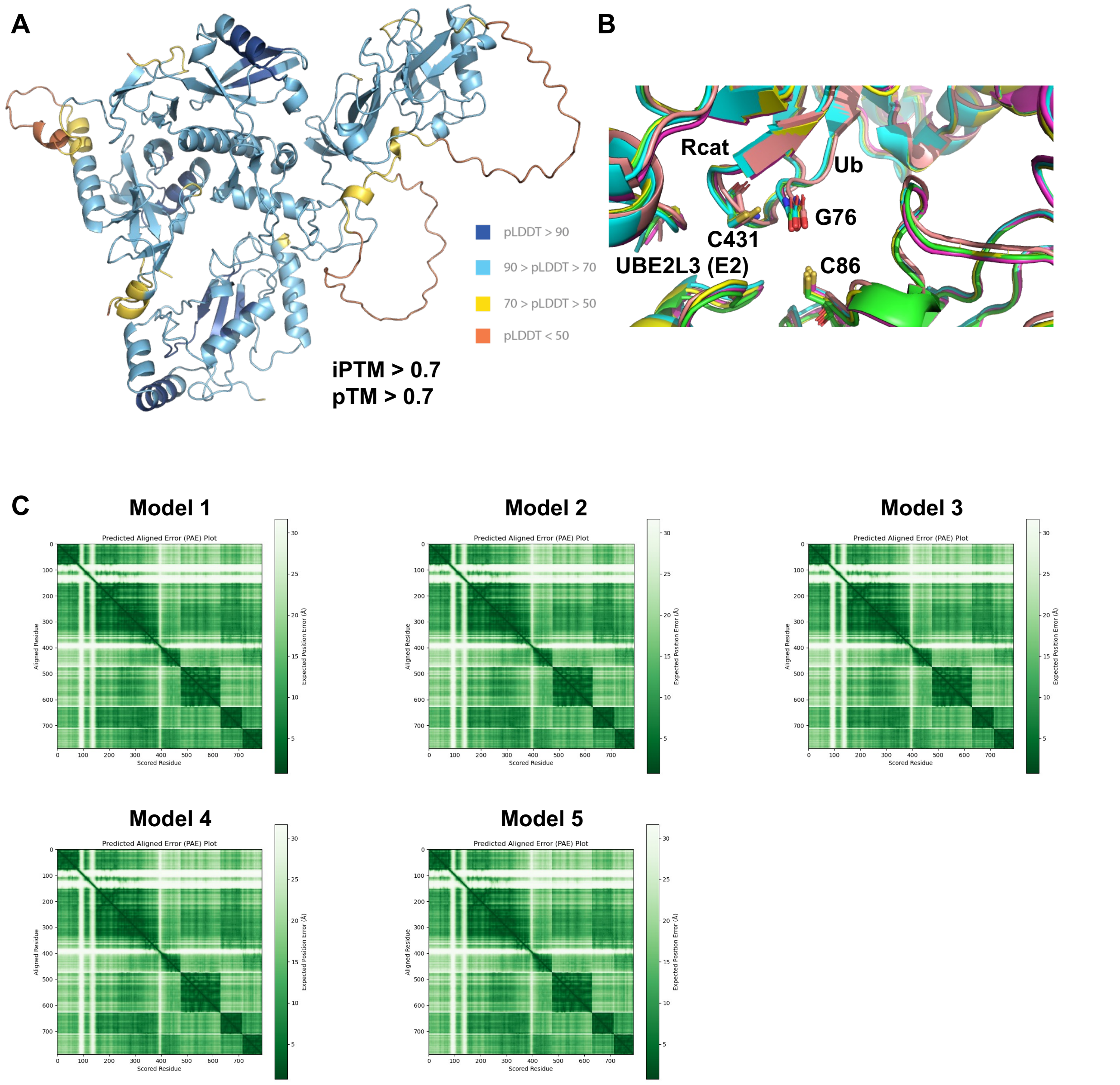

Supplementary Figure 3: AlphaFold 3 predicts the model of the complex with high confidence. (A) Model of phosphorylated parkin with bound allosteric phospho-ubiquitin and ubiquitin-charged UBE2L3 colored according to pLDDT scores (local confidence). (B) Zoom on the overlay of the five models showing the parkin/E2 interface with the catalytic cysteine on UBE2L3 and on the Rcat domain as well as the C-terminal glycine of the ubiquitin in stick representation. (C) Predicted aligned error (PAE) plots for the five predicted models. The PAE plot can be seen as a model for global confidence in the relative position of all pairs of residues.

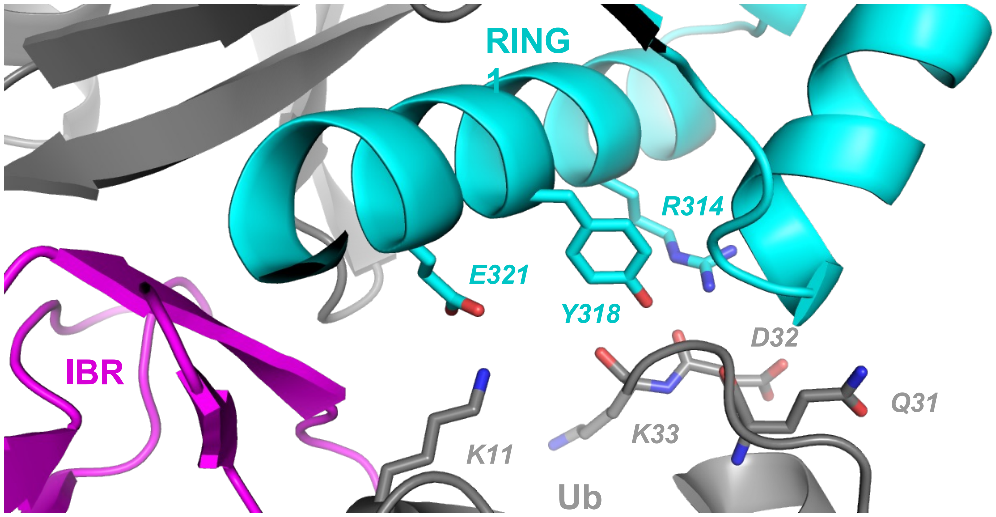

Supplementary Figure 4: RING1-Ub interface. The RING1 domain of parkin maintains the ubiquitin charged onto E2 in position for the transthiolation reaction through interactions between R314 and Y318 with the backbone of respectively Ub Q31 and D32, and K33, as well as E321 with Ub K11. This is in agreement with a previous study (Kumar et al., Nat Struct Mol Biol 2017, 24, 475-483) that reported Y318A and E321A mutants were unable to make ubiquitin chains and ubiquitinate Miro and that E321A pParkin:pUb has reduced affinity for E2~Ub.

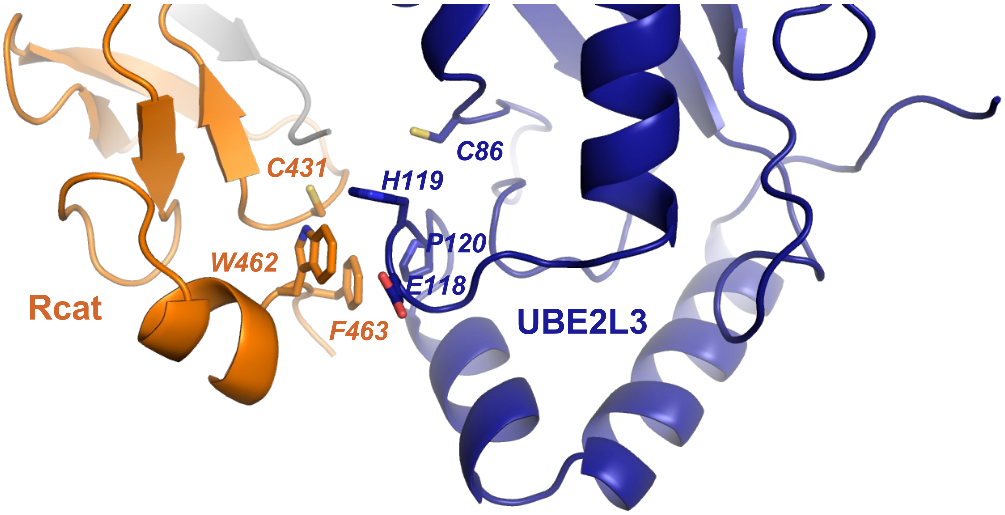

Supplementary Figure 5: Rcat-UBE2L3 interface. Aromatic residues (W462 and F463) at the C-terminal end of the Rcat domain, close to the catalytic cysteine C431, which are involved in RING0-Rcat interactions in inactive parkin, stack against a loop of UBE2L3 (res.118-120) to maintain E2 and Rcat proximity for the ubiquitin transfer.
